# Adrenomedullin-RAMP2 System Modulates Inflammation and Tissue Repair in Experimental Autoimmune Uveitis Via T-Cell and M2 Macrophage Regulation

**DOI:** 10.1167/iovs.66.6.12

**Published:** 2025-06-04

**Authors:** Yorishige Matsuda, Megumu Tanaka, Yunlu Zhao, Shinji Kakihara, Ken Hoshiyama, Takayuki Sakurai, Akiko Kamiyoshi, Yuka Ichikawa-Shindo, Hisaka Kawate, Yan Zhang, Qianqian Guo, Peixuan Li, Jiake Li, Jun Duan, Marina Hayashi, Hideki Sanjo, Toshinori Murata, Takayuki Shindo

**Affiliations:** 1 Department of Cardiovascular Research, Shinshu University School of Medicine, Matsumoto, Nagano, Japan; 2 Department of Ophthalmology, Shinshu University School of Medicine, Matsumoto, Nagano, Japan; 3 Department of Molecular and Cellular Immunology, Shinshu University School of Medicine, Matsumoto, Nagano, Japan

**Keywords:** adrenomedullin, EAU, macrophages, T cells, tissue repair

## Abstract

**Purpose:**

Adrenomedullin (AM), a peptide produced by various cells, exerts diverse physiological effects and is regulated by receptor activity-modifying proteins (RAMP2 and 3). Experimental autoimmune uveitis (EAU) is a well-established model for studying human autoimmune uveitis. Hence, we investigated the pathophysiological roles of the AM-RAMP system in uveitis using an optimized EAU mouse model.

**Methods:**

Female wild-type (WT), AM knockout, RAMP2KO, and RAMP3KO mice were immunized with the human interphotoreceptor retinoid-binding protein. The expression of macrophage-related genes and inflammatory cytokines in the retina and spleen was analyzed using real-time polymerase chain reaction. EAU-induced WT mice received human recombinant AM; therapeutic effects were evaluated via clinical and histologic scores, quantification of T-cell and macrophage infiltration in the retina, and the number of splenic regulatory T cells (Tregs) and M2 macrophages using flow cytometry.

**Results:**

Compared with WT mice, EAU-induced AMKO and RAMP2KO mice had significantly increased retinal inflammatory cell infiltration and worsened clinical scores, whereas RAMP3KO mice did not. Proinflammatory cytokine expression was suppressed in the retina of EAU-induced WT mice that received AM. However, anti-inflammatory cytokine expression was upregulated compared with that in the vehicle group. Additionally, there was reduced retinal infiltration of T cells and macrophages, leading to improved clinical and histologic scores. AM administration also suppressed EAU-induced splenomegaly and increased the number of Tregs and M2 macrophages, possibly contributing to resolving inflammation.

**Conclusions:**

AM exerts an anti-inflammatory effect in uveitis by activating Tregs and M2 macrophages through RAMP2. Its administration is a potential adjunctive therapy for uveitis.

Uveitis is a sight-threatening ocular inflammatory disorder characterized by retinal vascular leakage, inflammatory lesions, and macular edema,^1^^–^^4^ accounting for 5% to 20% of legal blindness in the United States and Europe and approximately 25% of blindness in developing nations.^5^^,^^6^ Etiologically, it can be classified as infectious and noninfectious, with noninfectious uveitis (NIU) being the most common, which tends to recur once triggered and often has a chronic course.^7^^,^^8^ The etiopathogenesis of NIU remains unclear; however, a combination of polygenic and environmental factors may contribute to the pathogenesis by disrupting the balance between inflammatory and regulatory immune mechanisms.^9^^,^^10^

Notably, experimental autoimmune uveitis (EAU) is an animal model that is widely used because it replicates many clinicopathologic features of human autoimmune uveitis.^11^ The etiology of EAU closely resembles human uveitis based on an autoimmune nature, in which patients show immunologic responses of circulating lymphocytes to retinal antigens. In the murine model, EAU is triggered by the systemic activation of ocular-specific CD4-positive T cells, which are typically located in or around the photoreceptor segments.^12^ EAU is mediated by pathologic T cells, particularly T helper 1 (Th1) and Th17 cells, which are derived from naive T cells in response to exposure to proinflammatory cytokines.^13^ This model facilitates the investigation of the underlying mechanisms of uveitis and the evaluation of potential therapeutics for ocular inflammatory diseases.^14^^–^^16^ However, current treatment options, primarily systemic corticosteroids, are limited by systemic side effects. Additionally, they can lead to ocular complications such as cataracts and glaucoma.^17^^–^^23^ Furthermore, immunosuppressive drugs help to control the disease with lower corticosteroid doses. However, drugs such as cyclosporine may cause side effects that are not associated with steroid use, such as proximal tubular damage.^24^^–^^27^ Additionally, biological agents, including monoclonal antibodies, are a relatively new therapeutic class; however, achieving stable anti-inflammatory effects with these agents may require several months. Moreover, some patients may be refractory to these treatments or encounter adverse drug reactions.^28^^–^^34^ Notably, biologics may increase the risk of opportunistic infections when combined with corticosteroids or immunosuppressive drugs.^35^

Adrenomedullin (AM) is a potential therapeutic avenue that is underexplored in uveitis. It is a bioactive peptide isolated from human pheochromocytoma.^36^ It belongs to the calcitonin superfamily and acts through the calcitonin receptor–like receptor (CLR), regulated by three receptor activity-modifying proteins (RAMPs): RAMP1, RAMP2, and RAMP3. CLR-RAMP2 and CLR-RAMP3 complexes have a high affinity for AM.^37^ Various physiological effects are attributable to AM, including anti-inflammatory, antiapoptotic, and antioxidative stress effects,^38^^–^^41^ which contribute to homeostasis across various organs and tissues,^39^^,^^42^ including the eyes.^43^

We previously reported that AM ameliorates pathologic angiogenesis and hyperpermeability in the retina in ophthalmic disease models of diabetic retinopathy^44^ and retinal vein occlusion (RVO).^45^ Additionally, we reported that AM improves the pathogenesis of neovascular age-related macular degeneration (AMD).^46^ In the RVO model, AM and RAMP2 knockout (KO) mice had more severe phenotypes, such as retinal hemorrhage and vein dilatation with tortuousness, than the other mice. Furthermore, AM administration reduced inflammation and improved vascular reperfusion in the RVO-induced wild-type (WT) mice.^45^

In the AMD model, the AM KO and RAMP2 KO mice showed increased laser-induced choroidal neovascularization, fibrosis, and inflammation compared with those in the WT mice. Notably, exogenous AM administration suppressed these pathologic features and downregulated fibrosis-related molecules.^46^ These findings highlight the potential of the AM-RAMP2 system as a novel therapeutic target for RVO and AMD.

We hypothesized that the AM-RAMP2 system could have therapeutic value in uveitis, considering that the condition involves abnormal autoimmune responses and shares inflammatory and vascular permeability features with RVO and AMD. However, the specific effects of AM on local inflammation in uveitis, including its role in T-cell differentiation and macrophage recruitment, remain unclear. Herein, we optimized experimental conditions to enhance the reproducibility of the EAU model and conducted investigations, including EAU-induced AM, RAMP2, and RAMP3 KO mice and exogenous AM administration in EAU-induced WT mice, to investigate the therapeutic potential of the AM-RAMP2 and AM-RAMP3 systems for autoimmune uveitis.

## Methods

### Animals

Eight-week-old female C57BL/6J WT mice were obtained from a supplier of laboratory animals (Charles River Laboratories Japan, Kanagawa, Japan). The AM,^47^ RAMP2,^48^ and RAMP3^49^ KO mice were previously generated by our group. AM KO mice were generated using C57BL/6J and 129 hybrids, RAMP2 KO mice were generated using C57BL/6J, and RAMP3 KO mice were generated using C57BL/6J and 129 hybrids but crossed back to the C57BL/6J strain after five generations. Therefore, it is not appropriate to simply compare the WT (+/+) in the two KO mouse lines. Each KO mouse must be compared to the WT (+/+) in its respective littermate. To make this distinction clear, we have labeled the WT model “+/+” as opposed to “±” for the heterozygous model. However, as we previously reported that generating homozygous AM (−/−) and RAMP2 (−/−) mice is embryonically lethal because of systemic edema and hemorrhage, which is primarily caused by abnormal vascular development,^47^ heterozygous AM (±) and RAMP2 (±) mice were used. In these mice, the affected gene expression is reduced to approximately half of that in WT mice.^48^ The following mice were used in this study: 8- to 10-week-old female AM (±), RAMP2 (±), RAMP3 (−/−), and WT mice, along with C57BL/6J WT mice that received systemic AM administration using osmotic pumps. All mice were bred and maintained under specific pathogen-free conditions, with controlled circadian rhythms, temperature, and humidity. The mice were anesthetized via intraperitoneal injection comprising an anesthetic that included 0.3 mg/kg medetomidine (Nippon Zenyaku Kogyo, Koriyama, Japan), 4.0 mg/kg midazolam (Astellas Pharma, Tokyo, Japan), and 5.0 mg/kg butorphanol (Meiji Seika Pharma, Tokyo, Japan). All experiments adhered to the ARVO Statement for the Use of Animals in Ophthalmic and Vision Research and our institutional guidelines. Furthermore, all animal handling procedures were approved by the Ethics Committee of Shinshu University School of Medicine (#024040, #024041).

### AM Administration in Mice

**Figure 1.**
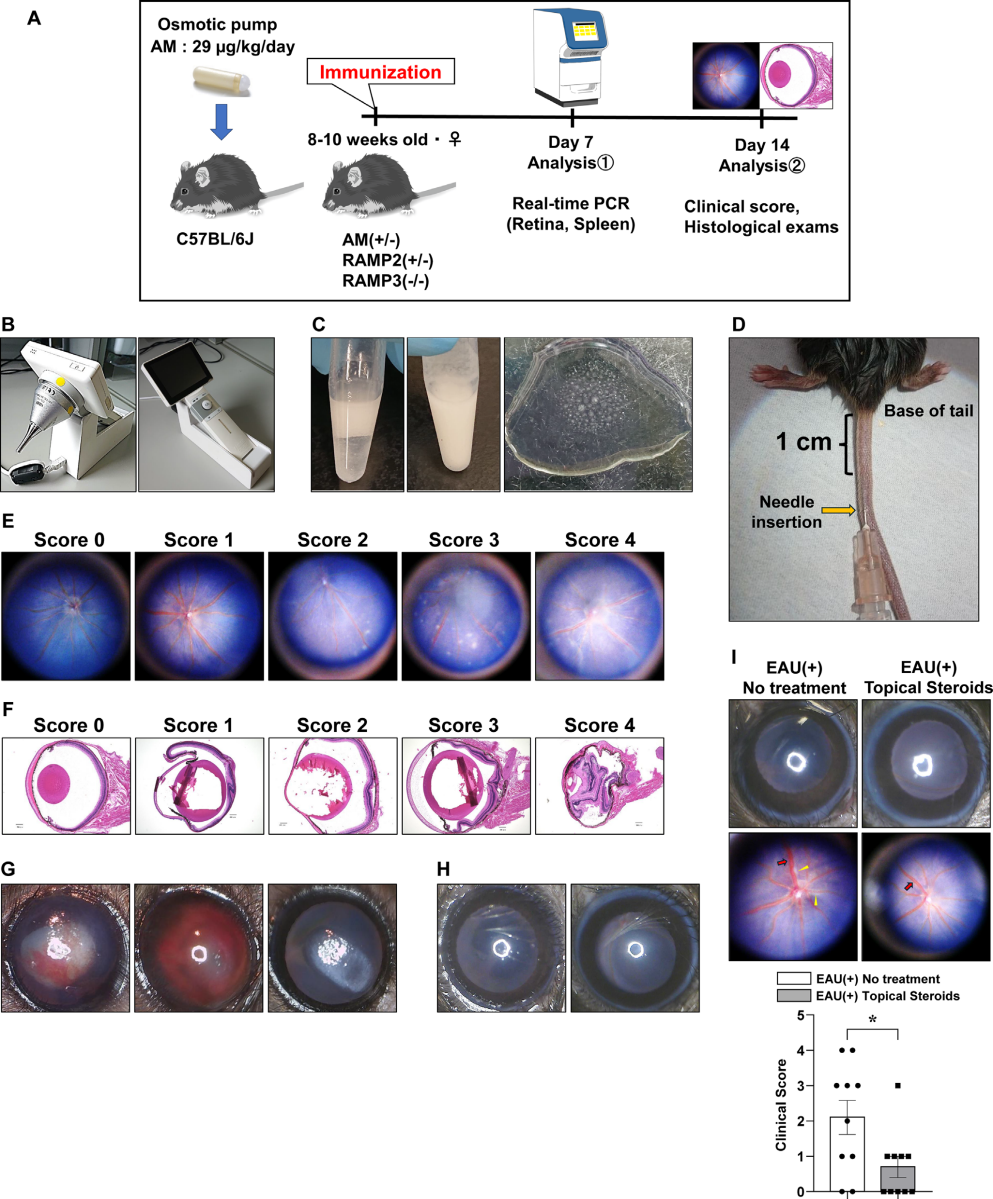
Protocol and optimization of the EAU model by fine-tuning various factors. (**A**) The timeline of immunization with hIRBP and administration of AM or PBS using an osmotic pump for C57BL/6J mice. Additionally, different sacrifice time points after immunization are shown. (**B**) Eyes are photographed using a portable camera on day 14 postimmunization. (**C**) To induce EAU, hIRBP is suspended in PBS and emulsified with an equal volume of CFA. Emulsion quality is satisfactory if the extruded emulsion does not separate and remains as droplets on a water surface for >2 hours. (**D**) Mice are immunized by injecting 80 µL of the emulsion mixture subcutaneously at approximately 1 cm from the base of the tail. Successful administration results in a white band discoloration at the injection site. (**E**) Fundus photographs representing a range of disease scores. (**F**) HE staining of the retinal section represents a range of disease scores. (**G**, **H**) Anterior ocular segment appearance of EAU before and after fine-tuning the reagent volume (**G**); the fundus or even pupil is obscured due to severe hyphema, mature cataract, or corneal opacity. After fine-tuning the reagent volume (**H**), a translucent appearance with the pupil and fundus is clearly visible. (**I**) Representative photographs and EAU clinical scores for mice in the no-treatment control (*n* = 10) and topical steroid treatment (*n* = 10) groups at 14 days after EAU induction. The clinical scores for EAU determined from the fundus photographs are indicated. *Yellow arrowheads*, retinal linear exudate; *red arrows*, retinal vessel engorgement. *Bars*: mean ± SEM. *P < 0.05, **P < 0.01, ***P < 0.001 (unpaired *t*-test).

Human AM (Peptide Institute, Osaka, Japan), dissolved in phosphate-buffered saline (PBS), was infused into subcutaneous tissues using osmotic pumps (Alzet; DURECT, Cupertino, CA, USA) immediately after EAU induction. The delivery rate was set at 29 µg/kg/d, according to the doses in our previous RVO^45^ and AMD^46^ models, where no adverse effects from AM treatment were observed, and the mice received AM for 7 or 14 days (Fig. 1A). The PBS-treated mice served as controls.

### Topical Endoscopy Fundus Imaging

The mice were anesthetized, as previously described, at 14 days post-EAU induction. The pupils were dilated using topical 0.5% phenylephrine and 0.5% tropicamide (Mydrin-P; Santen, Osaka, Japan). Eye gel (Scopisol; Senju, Osaka, Japan) was introduced into the cornea to facilitate contact with a digital medical scope (VersaCam; NIDEK, Aichi, Japan). The endoscope's position was adjusted by horizontally moving its tip against the gel-covered cornea. Subsequently, focus and illumination were adjusted, and the images of the fundus were obtained (Fig. 1B).^50^

### EAU Induction

EAU was induced in 8- to 10-week-old female mice using a subcutaneous injection of 200 µg of an amine-terminal peptide fragment (residues 1–20, GPTHLFQPSLVLDMAKVLLD) of human interphotoreceptor retinoid-binding protein (hIRBP) (MedChemExpress, Middlesex County, NJ, USA), which was emulsified in complete Freund's adjuvant (CFA) containing 2 mg/mL *Mycobacterium tuberculosis* H37Ra (Iwai Chemicals, Tokyo, Japan).^51^^–^^54^ Equal volumes of hIRBP and CFA were mixed thoroughly (approximately 2 minutes of continuous vortexing with 3- to 5-minute intervals between sets for 10–15 sets) until homogeneity was achieved. The final emulsion did not separate when dripped onto a water surface (Fig. 1C). This emulsion (80 µL in total) was injected subcutaneously at approximately 1 cm from the base of the tail (Fig. 1D). The fluid entry site showed a slight discoloration after successful administration. Concurrently, 1.2 µg pertussis toxin (PTX) (FUJIFILM Wako Pure Chemical Corporation, Osaka, Japan) was administered intraperitoneally.

### Scoring of EAU

Topical endoscopy fundus imaging was performed as described above. The EAU clinical score was graded based on the photographs by retinal specialists in a blinded manner, using a 0 to 5 scale to evaluate disease severity. This was based on previous reports with some modifications (Fig. 1E; Table 1).^55^^–^^61^

**Table 1.** The Clinical Score for Experimental Autoimmune Uveitis

Score	Description
Score 0	No evidence of inflammation
Score 1	Retinal vessel engorgement without exudate
Score 2	Retinal vessel engorgement and/or spotted exudate of **fewer** **than** **five** **spots**
Score 3	Retinal vessel engorgement and/or spotted exudate of **more than** **five** **spots**
Score 4	Retinal vessel engorgement and/or **linear exudates** along with vasculitis
Score 5	Exudative retinal detachment or subretinal hemorrhage

### Histologic Examination

The mice were euthanized by cervical dislocation 14 days post-EAU induction for histologic assessment. Eyeballs were removed, fixed in 4% paraformaldehyde, and processed into paraffin-embedded sections (5 µm thick) along the papillary–optic nerve plane. The sections were stained with hematoxylin and eosin (HE) and examined using a microscope (BZX710; Keyence, Osaka, Japan). Next, HE-stained eye sections were magnified 4× and photographed to include the entire eye. The EAU severity was scored for each eye using a 0 to 4 grading, based on previous reports with some modifications (Fig. 1F; Table 2),^55^^–^^61^ and subsequently quantified using a BZ analyzer (Keyence). For CD3 or F4/80 staining, the number of positive cells was counted in a 400× field of view. The data were obtained in a blinded manner.

**Table 2.** The Histologic Score for Experimental Autoimmune Uveitis

Score	Description
Score 0	No retinal destruction and normal retinal architecture
Score 1	**One retinal fold** without the presence of retinal detachment
Score 2	**Two retinal folds** or one focal retinal detachment*
Score 3	More than **two focal retinal detachments**
Score 4	**Diffuse** retinal detachment

* Two retinal folds are equivalent to one focal retinal detachment.

### Quantitative Reverse Transcription Polymerase Chain Reaction Analysis

cDNA was synthesized from total RNA, which was extracted from the retina or spleen. This was followed by quantitative reverse transcription polymerase chain reaction (qRT-PCR) to quantify relative mRNA levels using specific primers. The detailed methods of the cDNA synthesis, primer design, and qRT-PCR conditions are provided in the Supplementary Methods. PCR primers are listed in Table 3.

**Table 3.** Primers Used for Real-Time PCR

Primer Name	Primer Type	Primer Sequence
Adm	Forward	5′-GGACACTGCAGGGCCAGAT-3′
(adrenomedullin)	Reverse	5′-GTAGTTCCCTCTTCCCACGACTTA-3′
Calcrl	Forward	5′-AGGCGTTTACCTGCACACACT-3′
(CLR)	Reverse	5′-CAGGAAGCAGAGGAAACCCC-3′
Ramp2	Forward	5′-ACTGAGGACAGCCTTGTGTCAAA-3′
(RAMP2)	Reverse	5′-CCTTGACAGAGTCCATGCAACTC-3′
Ramp3	Forward	5′-AAAGCCTTCGCTGACATGATG-3′
(RAMP3)	Reverse	5′-ATCTCGGTGCAGTTAGTGAAGCT-3′
Il1b	Forward	5′-CTACAGGCTCCGAGATGAACAAC-3′
(IL-1b)	Reverse	5′-TCCATTGAGGTGGAGAGCTTTC-3′
Il4	Forward	5′-TCATCGGCATTTTGAACGAG-3′
(IL-4)	Reverse	5′-CGAGCTCACTCTCTGTGGTG-3′
Il6	Forward	5′-CCCAATTTCCAATGCTCTCC-3′
(IL-6)	Reverse	5′-TGAATTGGATGGTCTTGGTCC-3′
Il10	Forward	5′-CAGCCGGGAAGACAATAACTG-3′
(IL-10)	Reverse	5′-CCGCAGCTCTAGGAGCATGT-3′
Il13	Forward	5′-CCTCTGACCCTTAAGGAGCTT-3′
(IL-13)	Reverse	5′-ATGTTGGTCAGGGAATCCAG-3′
Il17a	Forward	5′-TGGCGCAAAAGTGAGCTCCAGAAG -3′
(IL-17A)	Reverse	5′-CGGCACTGAGCTTCCCAGATCAC -3′
Ifng	Forward	5′-TCAAGTGGCATAGATGTGGAAGAA-3′
(IFN-g)	Reverse	5′-AGAGATAATCTGGCTCTGCAGGAT-3′
Tnf	Forward	5′-ACGGCATGGATCTCAAAGAC-3′
(TNF-a)	Reverse	5′-AGATAGCAAATCGGCTGACG-3′
Ccl2	Forward	5′-GCAGTTAACGCCCCACTCA-3′
(MCP-1)	Reverse	5′-CCTACTCATTGGGATCATCTTGCT-3′
Cd3e	Forward	5′-ACGATGCCGAGAACATTGAATA-3′
(CD3e)	Reverse	5′-CATGCTTCTGAGGCAGCTCTT-3′
Cd4	Forward	5′-AGGAAGTGAACCTGGTGGTG-3′
(CD4)	Reverse	5′-CTCCTGCTTCAGGGTCAGTC-3′
Cd8a	Forward	5′-CTTCCAGAACTCCAGCTCCAAA-3′
(CD8)	Reverse	5′-GTGTCCCTCATGGCAGAAAAC-3′
Foxp3	Forward	5′-CTGCATCGTAGCCACCAGTA-3′
(Foxp3)	Reverse	5′-TGGAAGAACTCTGGGAAGGA-3′
Itga	Forward	5′-CTGGATAGCCTTTCTTCTGCTG-3′
x (CD11c)	Reverse	5′-GCACACTGTGTCCGAACTC-3′
Mrc1	Forward	5′-TCAGCTATTGGACGCGAGGCA-3′
(CD206)	Reverse	5′-TCCGGGTTGCAAGTTGCCGT-3′
Adgre1	Forward	5′-GATGAATTCCCGTGTTGTTGGT-3′
(F4/80)	Reverse	5′-ACATCAGTGTTCCAGGAGACACA-3′
Nos2	Forward	5′-ATGTGGCTACCACATTGAAGAAGC-3′
(iNOS)	Reverse	5′-AAGACTGCACCGAAGATATCTTCATG-3′

### Transcriptome Analysis and Data Processing

The transcriptome analysis was conducted using a mouse Clariom S array (Thermo Fisher Scientific, Waltham, MA, USA). The complete description of this analysis and data processing are provided in Supplementary Methods.

### Flow Cytometry

Single-cell suspensions from spleens were prepared, followed by viability staining and Fc receptor blocking. Subsequently, fluorochrome-conjugated antibodies were used to stain specific cell populations for analysis using fluorescence-activated cell sorting (FACS). Detailed procedures are provided in Supplementary Methods. The regents and fluorochrome-conjugated antibodies used are listed in Table 4. Gating was performed as shown in Supplementary Figs. S1A, S2.

**Table 4.** Antibodies Used for Fluorescence-Activated Cell Sorting

Name of Antibody	RRID	Manufacturer, Catalog No., and/or Name of Individual Providing the Antibody	Host Species, Monoclonal or Polyclonal
F4/80	AB_2563102	BioLegend (San Diego, CA, USA), #123137	Rat, monoclonal
CD11b	AB_2629529	BioLegend (San Diego, CA, USA), #101263	Rat, monoclonal
CX3CR1	AB_2565707	BioLegend (San Diego, CA, USA), #149025	Mouse, monoclonal
CD4	AB_2563054	BioLegend (San Diego, CA, USA), #100548	Rat, monoclonal
CD11c	AB_2562414	BioLegend (San Diego, CA, USA), #117339	American hamster, monoclonal
Ly6c	AB_2565852	BioLegend (San Diego, CA, USA), #128041	Rat, monoclonal
MHCⅡ	AB_313320	BioLegend (San Diego, CA, USA), #107605	Rat, monoclonal
CD45	AB_893340	BioLegend (San Diego, CA, USA), #103131	Rat, monoclonal
CD206	AB_2562247	BioLegend (San Diego, CA, USA), #141719	Rat, monoclonal
SiglecF	AB_2750236	BioLegend (San Diego, CA, USA), #155507	Rat, monoclonal
Ly6G	AB_10643269	BioLegend (San Diego, CA, USA), #127622	Rat, monoclonal
iNOS	AB_2572641	eBioscience (Carlsbad, CA, USA), #12592080	Rat, monoclonal
CD3	AB_394595	BD Bioscience (Franklin Lakes, NJ, USA), #553061	American hamster, monoclonal
Foxp3	AB_465936	eBioscience (Carlsbad, CA, USA), #12577382	Rat, monoclonal
Anti-CD16/CD32 (Mouse Fc Block) (2.4G2)	AB_394656	BD Bioscience (Franklin Lakes, NJ, USA), #553142	Rat, monoclonal

RRID, Research Resource Identifier.

### Statistical Analysis

Statistical analysis was performed using GraphPad Prism 9.3.1 (GraphPad Software, La Jolla, CA, USA). The values are expressed as mean ± SEM. A *t*-test and one-way analysis of variance, followed by Dunnett's test, were used to determine significance levels. Statistical significance was set at *P* < 0.05.

## Results

### Optimization of the EAU Model by Fine-Tuning Various Factors

EAU is a widely used animal model of human autoimmune uveitis; however, the quantity of reagent and administration methods used to induce inflammation, along with the evaluation approaches for inflammatory findings, are significantly varied across studies. Thus, we investigated the optimal protocol for effective EAU induction in C57BL/6J mice, drawing references from previous reports.^62^

Initially, EAU was induced using 250 µg hIRBP and emulsified in CFA containing 2.5 mg/mL *M.* *tuberculosis* H37Ra. The mixed hIRBP–CFA emulsion (100 µL) was subcutaneously injected, followed by 1.5 µg intraperitoneal PTX. However, this approach approximately led to a 50% mortality rate among the mice, and those that survived had excessive anterior segment inflammation, which precluded fundus examination because of issues such as anterior chamber hemorrhage, posterior iris synechiae, mature cataracts, and corneal opacity (Fig. 1G).

Notably, the EAU incidence and inflammation severity are highly variable across different studies using the C57BL/6 mouse strain. The variability is attributable to inconsistent susceptibility, leading to induction uncertainties.^63^^–^^66^ Various factors, particularly the hIRBP1-20 peptide and PTX doses, can influence EAU incidence.^67^ In this study, we used a vortex device to mix hIRBP and CFA, which enhanced both speed and ease compared with the conventional method of manually mixing two syringes using a three-way stopcock.^62^

The injection sites for hIRBP administration also varied across studies, including the back, neck, thighs, flanks, and footpad.^68^^–^^70^ We selected tail injections to avoid subtle differences resulting from skin pinching and injection site variances.

Reagent volumes were fine-tuned, including reducing hIRBP to 200 µg, *M.* *tuberculosis* H37Ra in CFA to 2 mg/mL, and PTX to 1.2 µg, each by 20%. Consequently, anterior segment inflammation was no longer observed, allowing for clear visualization of the fundus (Fig. 1H).

However, the model was unsuitable for reflecting clinically observed uveitis when posterior segment inflammation was too severe for treatment, even if the anterior segment was clear and the fundus was visible. Therefore, we confirmed that this model was also responsive to treatment, as posterior segment inflammation was somewhat mitigated by topical steroids (Sanbetason; Santen, Osaka, Japan) (Fig. 1I).

Additionally, the scoring system in our model for assessing the degree of inflammation was based on specific inflammatory findings (rather than vague descriptors), reducing examiner subjectivity (Tables 1 and 2). Experiments were conducted using the EAU model refined under these conditions.

### AM (±) Mice Exhibited More Pronounced Inflammatory Findings

**Figure 2.**
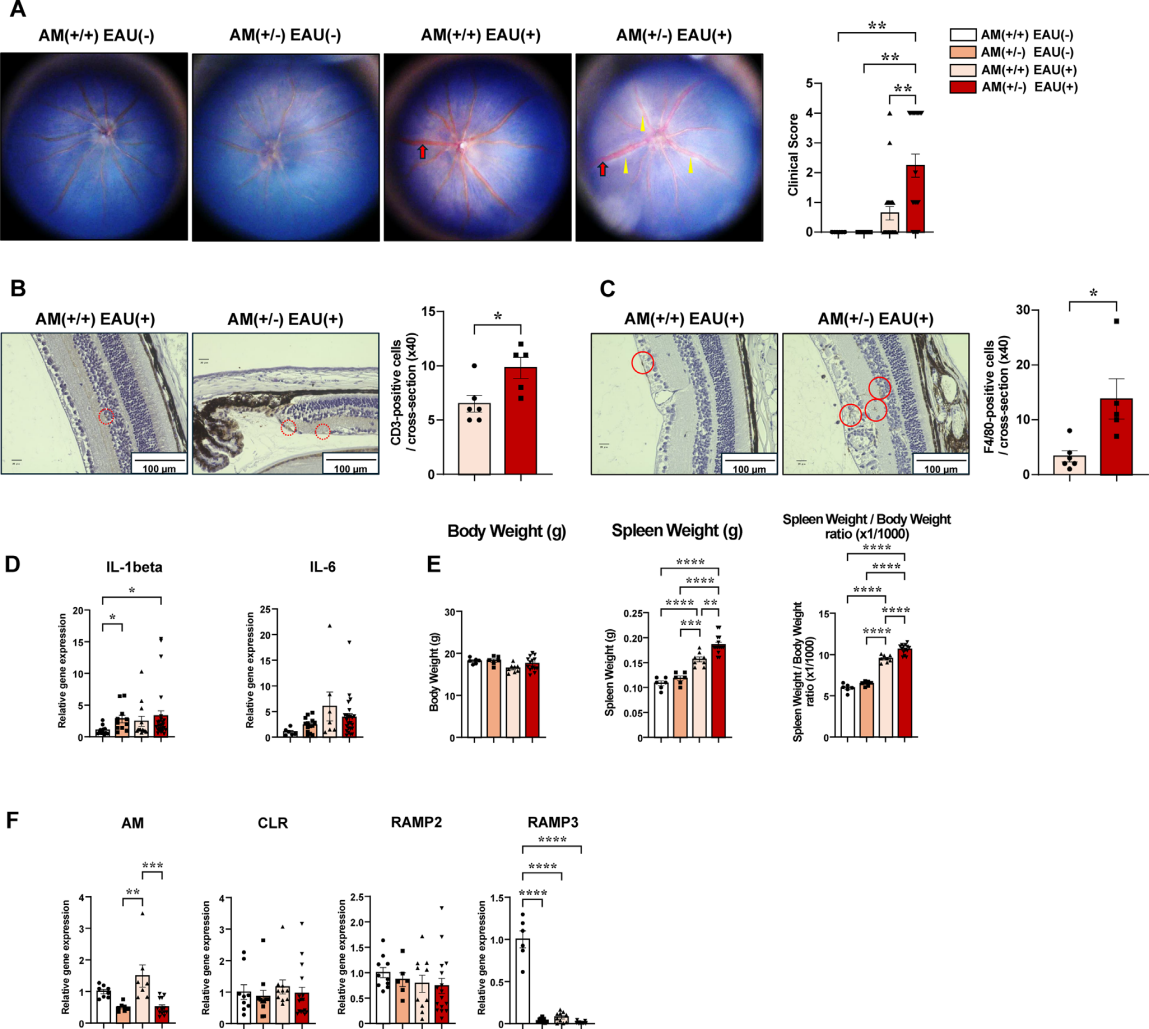
AM (±) mice exhibited more pronounced inflammatory findings. (**A**) Representative fundus photographs and EAU clinical scores for mice in the AM (+/+) (*n* = 18) and AM (±) (*n* = 23) groups at 14 days after EAU induction. Naive mice (*n* = 6) were similarly examined. The clinical score for EAU determined from the fundus photographs is indicated. *Yellow arrowheads*, retinal linear exudate; *red arrows*, retinal vessel engorgement. (**B**, **C**) Staining for immunohistochemical analysis of CD3-positive T-cell (**B**) and F4/80-positive macrophage (**C**) infiltration at 14 days following EAU induction. The number of immune cells per retina in one cross section of the AM (+/+) (*n* = 6) and AM (±) (*n* = 5) mice was counted. *Red dot circles*, CD-3 positive T cells; *red circles*, F4/80-positive macrophages. (**D**) Quantitative reverse transcription PCR analysis of inflammation-related genes (IL-1β and IL-6) in retinas on day 7 after immunization (*n* = 6–24). (**E**) Body weight, spleen weight, and the spleen weight/body weight ratio (×1/1000) of control (*n* = 6), AM (+/+) (*n* = 8), and AM (±) (*n* = 14) mice after EAU induction. (**F**) Quantitative reverse transcription PCR analysis of AM and AM-related receptor genes in spleens on day 7 after immunization (*n* = 6–17). *Bars*: mean ± SEM. *P < 0.05, **P < 0.01, ***P < 0.001, ****P < 0.0001 (unpaired *t*-test or one-way analysis of variance).

We examined heterozygous AM (±) and AM (+/+) mice to clarify the pathophysiologic role of endogenous AM in uveitis. In the absence of EAU induction, neither AM (+/+) nor AM (±) mice showed any inflammatory change in the fundus. After EAU induction, both showed mild inflammation, including vascular engorgement and dilation. Additionally, AM (±) mice developed linear exudative lesions. Consequently, the clinical score in AM (±) mice was significantly higher than that in AM (+/+) mice (Fig. 2A). Furthermore, the number of CD3-positive T cells and F4/80-positive macrophages infiltrating the retina of AM (±) mice was significantly higher than that in the AM (+/+) mice (Figs. 2B, 2C). The expression of inflammatory cytokines (IL-1β and IL-6) in the retina tended to increase in AM (±) mice with and without EAU (Fig. 2D). Furthermore, the increase in spleen weight (along with splenomegaly) in EAU was significantly greater in the AM (±) mice than in the AM (+/+) mice (Fig. 2E and Supplementary Fig. S3). These exacerbated inflammatory findings suggest that AM considerably contributes to alleviating EAU-induced inflammation.

Additionally, the RAMP3 gene expression in the spleen showed near-complete depletion following either KO or EAU induction, regardless of the genotype. AM expression was halved in the heterozygous KO mice with or without inflammation, and the RAMP2 expression did not decrease or differ among groups, regardless of the presence of inflammation or genotype (Fig. 2F).

### RAMP2 (±) Mice Exhibited More Pronounced Inflammatory Findings

**Figure 3.**
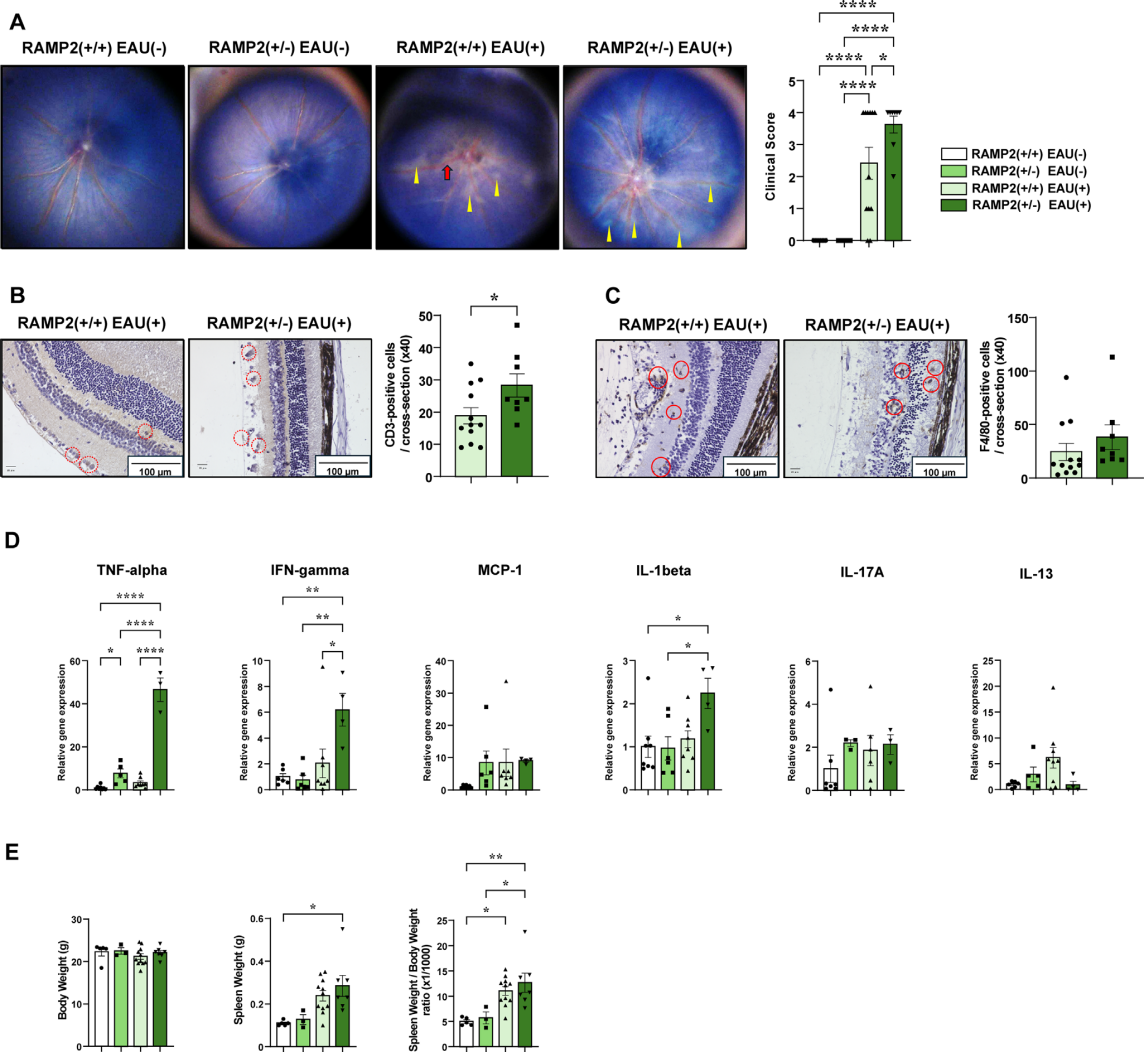
RAMP2(±) mice exhibited more pronounced inflammatory findings. (**A**) Representative fundus photographs and EAU clinical scores for mice in the RAMP2 (+/+) (*n* = 12) and RAMP2 (±) (*n* = 8) groups at 14 days after EAU induction. Naive mice (*n* = 6) were similarly examined. The clinical score for EAU determined from the fundus photographs is indicated. *Yellow arrowheads*, retinal linear exudate; *red arrows*, retinal vessel engorgement. (**B**, **C**) Staining for immunohistochemical analysis of CD3-positive T-cell (**B**) and F4/80-positive macrophage (**C**) infiltration at 14 days after EAU induction. The number of immune cells per retina in one cross section of the RAMP2 (+/+) (*n* = 12) and RAMP2 (±) (*n* = 8) mice was counted. *Red dot circles*, CD-3 positive T cells; *red circles*, F4/80-positive macrophages. (**D**) Quantitative reverse transcription PCR analysis of inflammation-related genes (TNF-α, IFN-γ, monocyte chemoattractant protein [MCP] 1, IL-1β, IL-6, and IL-10) in the retinas on day 7 after immunization (*n* = 3–8). (**E**) Body weight, spleen weight, and the spleen weight/body weight ratio (×1/1000) of control (*n* = 3–5), RAMP2 (+/+) (*n* = 11), and RAMP2 (±) (*n* = 7) mice after EAU induction. *Bars*: mean ± SEM. *P < 0.05, **P < 0.01, ***P < 0.001, ****P < 0.0001 (unpaired *t*-test or one-way ANOVA).

The EAU model in the AM (±) group revealed that RAMP2 significantly contributes to EAU pathogenesis among the RAMP sub-isoforms of AM. To verify this, we examined the importance of endogenous RAMP2 using RAMP2 (±) mice. Notably, both RAMP2 (+/+) and RAMP2 (±) mice had significantly increased clinical scores, with RAMP2 (±) having a significantly higher score than that of the RAMP2 (+/+) mice (Fig. 3A).

Furthermore, significantly more CD3-positive T cells infiltrated the retinas of the RAMP2 (±) mice than that of RAMP2 (+/+) mice (Fig. 3B). Similarly, F4/80-positive macrophages tended to increase more among the RAMP2 (±) mice than in the RAMP2 (+/+) mice (Fig. 3C). In RAMP2 (±) mice with EAU, the expression of inflammatory cytokines in the retina significantly increased, and certain markers tended to be elevated, even in the absence of EAU. Only IL-13 expression tended to be higher in the RAMP2 (+/+) mice (Fig. 3D). Additionally, the increase in spleen weight due to EAU was greater in the RAMP2 (±) mice than in the RAMP2 (+/+) mice, similar to the findings in AM (±) mice (Fig. 3E), suggesting that the AM-RAMP2 system plays a role in the inflammatory response and the splenomegaly caused by EAU.

### No Significant Difference Was Detected in Inflammatory Findings Between RAMP3 (+/+) and RAMP3 (−/−) Mice

**Figure 4.**
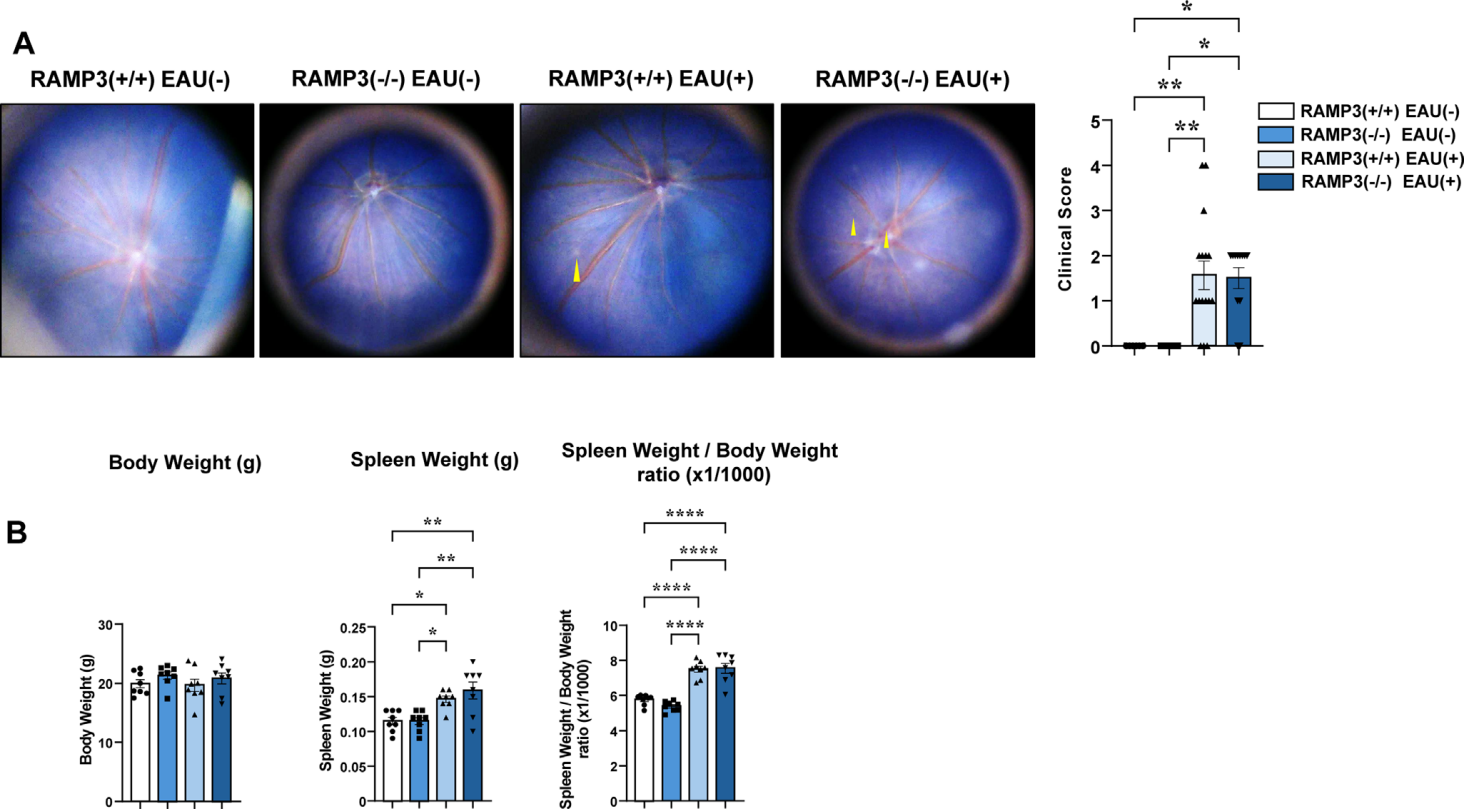
No significant difference was detected in inflammatory findings between RAMP3 (+/+) and RAMP3 (−/−) mice. (**A**) Representative fundus photographs and EAU clinical scores for mice in the RAMP3 (+/+) (*n* = 16) and RAMP3 (−/−) (*n* = 11) groups at 14 days after EAU induction. Naive mice (*n* = 6) were similarly examined. The clinical score for EAU determined from the fundus photographs is indicated. *Yellow arrowheads*, retinal linear exudate; *red arrows*, retinal vessel engorgement. (**B**) Body weight, spleen weight, and the spleen weight/body weight ratio (×1/1000) of control, RAMP3 (+/+), and RAMP3 (−/−) mice after EAU induction (*n* = 8).

RAMP3 expression in the spleen was significantly reduced in this model, suggesting that it does not significantly contribute to exacerbating EAU pathology. Subsequent experiments with RAMP3 (−/−) mice confirmed this; no significant differences were observed between the groups. After EAU induction, both RAMP3 (+/+) and RAMP3 (−/−) mice showed a significant increase in clinical scores; however, the two groups did not significantly differ (Fig. 4A). The EAU induced a significant increase in spleen weight; however, the increase did not significantly differ between RAMP3 (+/+) and RAMP3 (−/−) mice (Fig. 4B). These results confirm that, compared with the AM-RAMP2 system, the AM-RAMP3 system is less integral to the pathogenesis of EAU. However, the downregulation of RAMP3 gene expression caused by EAU induction may contribute to the lack of differences in outcomes between the two groups.

### Exogenous AM Administration in C57BL/6J Mice Significantly Improved Various Inflammatory Outcomes

The aforementioned experiments in genetically engineered mice demonstrated that the endogenous AM-RAMP2 system ameliorates uveitis pathogenesis in the EAU model by suppressing T-cell and macrophage infiltration into the retina and reducing proinflammatory cytokine expression in the retina. Accordingly, we administered exogenous AM to C57BL/6J WT mice to explore the potential therapeutic application of AM in uveitis.

**Figure 5.**
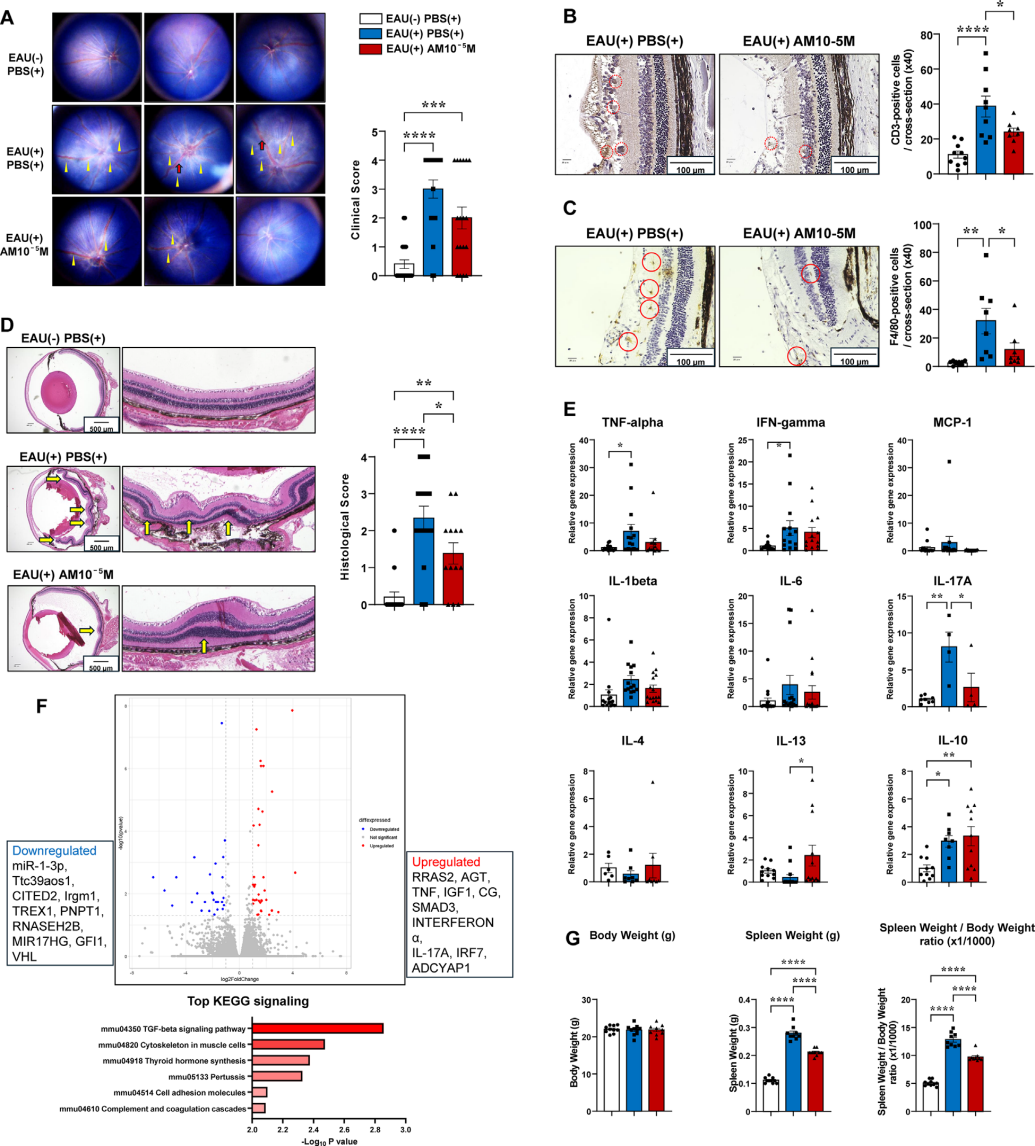
Exogenous adrenomedullin administration in C57BL/6J mice significantly improved various inflammatory outcomes. (**A**) Representative fundus photographs and EAU clinical score for mice in the control (*n* = 20), PBS-treated (*n* = 20), and AM-treated (*n* = 18) groups at 14 days after EAU induction. The clinical score for EAU determined from the fundus photographs is indicated. *Yellow arrowheads*, retinal linear exudate; *red arrows*, retinal vessel engorgement. (**B**, **C**) Staining for immunohistochemical analysis of CD3-positive T-cell (**B**) and F4/80-positive macrophage (**C**) infiltration at 14 days after EAU induction. The number of immune cells per retina in one cross section of the control (*n* = 10), PBS-treated (*n* = 9), and AM-treated (*n* = 8) mice was counted. *Red dot circles*, CD-3 positive T cells; *red circles*, F4/80-positive macrophages. (**D**) Representative histologic findings and EAU histologic scores for mice in the control (*n* = 15), PBS-treated (*n* = 15), and AM-treated (*n* = 13) groups at 14 days after EAU induction. Sections of the eye from control and EAU mice at 14 days after injection with hIRBP, stained with HE. *Yellow arrows*, retinal folds. *Scale bars*: 500 µm. (**E**) Quantitative reverse transcription PCR analysis of inflammation-related genes (TNF-α, IFN-γ, MCP-1, IL-1β, IL-6, and IL-10) in retinas on day 7 after immunization (*n* = 6–16). (**F**) Transcriptomic analysis of the EAU model in C57BL/6J mice identified candidate genes associated with the suppressive effects of AM. Volcano plots of the differentially expressed genes in 14-day samples by comparison between AM- and PBS-administered mice are shown (*n* = 2 in each). *Red spots* represent the upregulated genes, and *blue spots* represent the downregulated genes, with the fold change on the x-axis and *P* value on the y-axis. The *gray-colored region* represents the insignificantly differentially expressed genes. Gene Ontology (GO) enrichment histogram shows the top six most significantly enriched GO terms. (**G**) Body weight, spleen weight, and the spleen weight/body weight ratio (×1/1000) of control, PBS-, and AM-treated mice after EAU administration (*n* = 9–10). *Bars*: mean ± SEM. *P < 0.05, **P < 0.01, ***P < 0.001, ****P < 0.0001 (unpaired *t*-test or one-way ANOVA).

The results showed that the EAU induction significantly increased clinical scores; however, inflammatory findings, such as vasodilation and linear exudative spots, were alleviated in the AM-treated group, resulting in lower scores than those in the PBS-treated group (Fig. 5A). The infiltration of CD3-positive T cells and F4/80-positive macrophages was also significantly increased by EAU induction; however, the infiltration was significantly suppressed by AM treatment compared with that in the PBS-treated group (Figs. 5B, 5C).

In addition, HE staining revealed normal retinal structure in the absence of EAU. In the PBS-treated group, during EAU, retinal folds and exudative retinal detachment were observed; however, these findings considerably improved, and the histologic score was significantly suppressed in the AM-treated group (Fig. 5D). Furthermore, EAU induction elevated the expression levels of various inflammatory cytokines in the retina, and the AM treatment tended to suppress these compared with those in the PBS-treated group. Conversely, IL-13 and the anti-inflammatory cytokine IL-10 were significantly upregulated by AM treatment (Fig. 5E).

Moreover, transcriptome analysis of the retina 2 weeks post-EAU induction revealed enhanced activation of factors associated with cell adhesion and cytoskeletal remodeling, such as TGF-β and Smad3 (Fig. 5F). Furthermore, TGF-β and its downstream signaling molecule Smad3 suppressed inflammatory responses and prevented excessive inflammation, promoting tissue repair by inhibiting cell proliferation and extracellular matrix formation.^71^^,^^72^ This aligned with the improvement in exudative spots and serous retinal detachment secondary to increased vascular permeability, which led to significantly improved clinical and histological scores in the AM-treated group.

**Figure 6.**
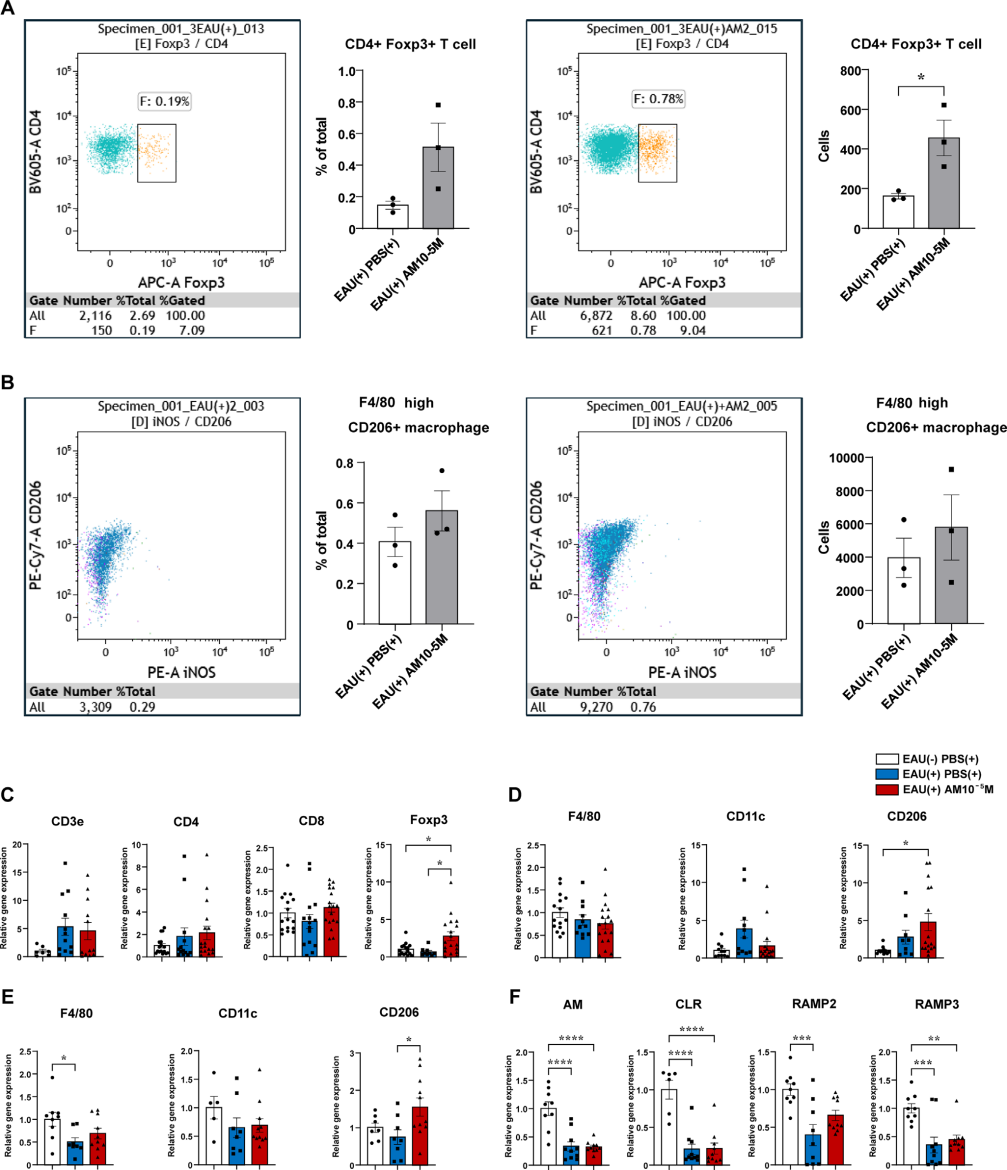
Further examinations for the mechanism of EAU suppression by the AM-RAMP2 system. (**A**, **B**) The absolute numbers and the percentage of the population of T regs (**A**) and M2 macrophages (**B**) in spleens of PBS-treated (*n* = 3) and AM-treated (*n* = 3) C57BL/6J mice in splenic flow cytometric analysis on day 7 after immunization. (**C**, **D**) Quantitative reverse transcription PCR analysis of T-cell-related (**C**) and macrophage-related genes (**D**) in retinas on day 7 after immunization (*n* = 7–18). (**E**, **F**) Quantitative reverse transcription PCR analysis of macrophage-related (**E**) and AM and AM-related receptor genes (**F**) in spleens on day 7 after immunization (*n* = 5–11). *Bars*: mean ± SEM. *P < 0.05, **P < 0.01, ***P < 0.001, ****P < 0.0001 (unpaired *t*-test or one-way ANOVA).

Furthermore, EAU significantly increased spleen weight; however, the AM-treated group showed significantly less weight gain compared with that in the PBS-treated group (Fig. 5G). Further examination of cell populations using splenic FACS revealed that Tregs (expressing Foxp3) and M2 macrophages (expressing CD206) were upregulated in the AM-treated group (Figs. 6A, 6B).

The increase in M2 macrophages, which promote inflammatory convergence and tissue repair, in the spleen of the AM-treated group indicates that AM has a systemic anti-inflammatory effect, consistent with the upregulated expression of Foxp3, a Treg marker, in the retina (Fig. 6C), as well as CD206, an M2 macrophage marker, in both the retina and spleen (Figs. 6D, 6E). These are also consistent with the observed improvement in inflammatory findings (scores) in the fundus and suppression of proinflammatory cytokine expression in the retina, along with reduced weight gain in the spleen. The increased expression of cytokines that activate M2 macrophages (IL-4 and, especially, IL-13) in the retina corroborated the anti-inflammatory effect.

## Discussion

In humans, elevated AM levels have been observed in eyes with ocular lesions such as proliferative diabetic retinopathy, glaucoma, retinitis pigmentosa, and uveitis.^73^ Furthermore, CLR and RAMP2 showed similar localization patterns to AM in the retina and stimulation of isolated retinas with AM increased immunoreactivity, suggesting that the AM pathway is also associated with retinal lesions.^74^ Notably, high glucose-induced human retinal endothelial cell migration and proliferation were previously inhibited by AM administration.^75^

Additionally, AM and its receptors are expressed in the iris ciliary body, where AM binding relaxes the iris sphincter muscle and reduces intraocular pressure.^76^^,^^77^ Reportedly, genetic mutations in RAMP2 in mice disrupt the AM-RAMP2/CLR and cAMP signaling pathways, leading to primary open-angle glaucoma through retinal ganglion cell death.^78^

Similarly, in our EAU model, exogenous AM administration significantly improved clinical scores and reduced inflammatory cell infiltration and cytokine expression compared with that in the vehicle group. This suggests that exogenous AM administration ameliorates the pathogenesis of uveitis. Furthermore, EAU induction strongly suppressed RAMP3 expression, whereas RAMP2 expression was unchanged. Exogenous AM administration tended to increase RAMP2 expression, whereas RAMP3 expression remained suppressed (Fig. 6F), suggesting that AM administration ameliorates the pathogenesis of uveitis via RAMP2 but not RAMP3. Notably, AM (±) and RAMP2 (±) mice exhibited worsening clinical scores, higher inflammatory cell infiltration, and elevated inflammatory cytokine expression compared to WT mice. In contrast, compared with the WT mice, the severity of the disease, including clinical scores in the RAMP3 (−/−) mice, did not significantly differ.

In addition, AM protects against experimental autoimmune encephalomyelitis,^79^ arthritis,^80^ and inflammatory bowel disease (IBD)^81^ by downregulating inflammatory cytokine production and Th1 response and inducing Tregs. In addition, AM-stimulated dendritic cells had lower levels of costimulator expression and proinflammatory cytokine release.^82^

In the EAU model, AM administration decreased the expression of IFN-γ and IL-17, cytokines produced by Th1 and Th17 cells, respectively. However, the expression of IL-13, a cytokine derived from Th2 cells, Foxp3, a Treg marker, and IL-10, an anti-inflammatory cytokine derived from Tregs, was increased. This shift in the balance of immune responses may transition the pathogenesis of uveitis from a proinflammatory to an anti-inflammatory state, thereby improving clinical outcomes.

Furthermore, we found that AM administration significantly enhanced M2 macrophage marker (CD206) expression in the retina and spleen. Splenic FACS analysis also showed an increase in M2 macrophages. Our findings also revealed that the TGF-β–Smad3 system was upregulated in the transcriptome analysis of the retina. Th2 cells secrete cytokines, such as IL-4 and IL-13, which bind to macrophage surface receptors and are transmitted via the STAT6 pathway,^83^ causing their differentiation into M2 macrophages. This activation is known as “alternative activation.”^84^ Notably, M2 macrophages secrete anti-inflammatory cytokines and growth factors, such as IL-10 and TGF-β, which promote anti-inflammatory responses, tissue repair, and regeneration and are crucial in regulating chronic inflammation.^85^ Autoimmune diseases, such as multiple sclerosis^86^ and type 1 diabetes,^87^ and chronic inflammatory diseases, such as Crohn disease,^88^ exhibit reduced M2 macrophage function, causing excessive inflammation and impaired tissue repair functions, aggravating the disease.

**Figure 7.**
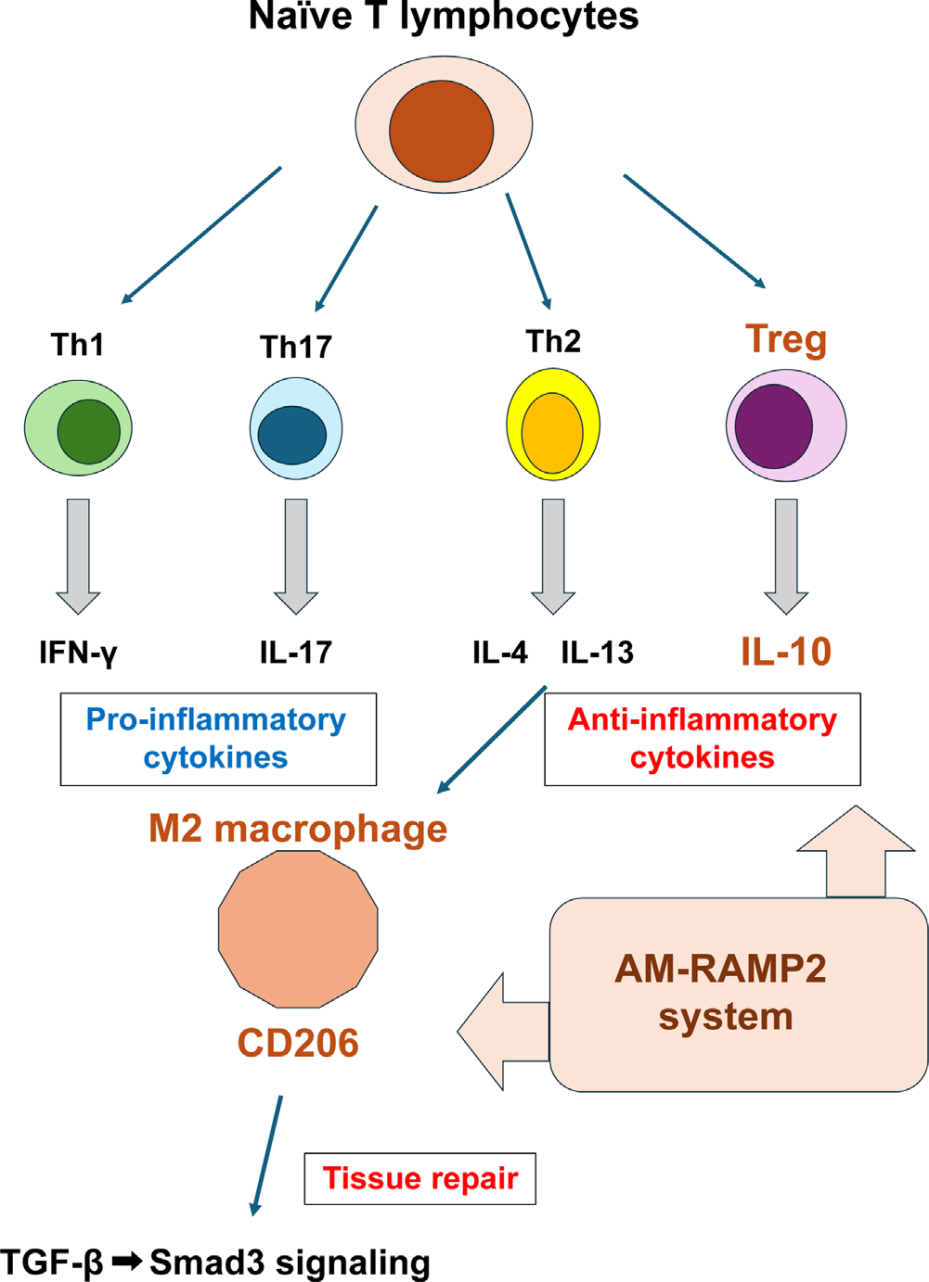
Schematic illustration showing the possible mechanism by which adrenomedullin promotes tissue repair and regeneration following the inflammatory response in EAU mice. In the EAU model, the AM-RAMP2 system is thought to exert anti-inflammatory effects and promote tissue repair by activating Tregs and M2 macrophages, thereby contributing to the improvement of disease pathology. Exogenous administration of AM reduced the expression of IFN-γ and IL-17, which are produced by Th1 and Th17 cells, respectively. In contrast, the expression of IL-13, a cytokine derived from Th2 cells, and IL-10, an anti-inflammatory cytokine derived from Tregs, was increased. Furthermore, M2 macrophages may promote tissue repair by activating the TGF-β–Smad3 signaling pathway.

Furthermore, the EAU, similar to other autoimmune disease models, showed significant pathologic changes due to AM-induced alterations in M2 macrophages. In the AM-treated group, the number of M2 macrophages increased, alongside a rise in inducible nitric oxide synthase (iNOS)–positive cell numbers and gene expression (Supplementary Figs. S1A, S1B). These iNOS-expressing cells, which lacked the macrophage marker F4/80, were likely granulocytes (possibly eosinophils) because of their relative size and complexity. Eosinophils, like M2 macrophages, are activated by IL-4 and IL-13,^89^^,^^90^ indicating that AM administration enhances the production of these cytokines. Furthermore, AM administration upregulates the IL-13 receptor α2 chain.^91^ Figure 7 summarizes the proposed mechanisms by which AM administration improves disease status in EAU. AM suppressed chronic inflammation, promoted tissue repair, and improved the pathology of uveitis by activating Tregs and M2 macrophages.

In the present study, we demonstrated that AM is a promising therapeutic target for preventing and treating autoimmune uveitis. As a bioactive peptide naturally present in the body, AM is believed to be safe and has fewer side effects compared to traditional immunosuppressants, which exert strong effects. In contrast, AM modulates immune function, potentially reducing side effects. Furthermore, the tissue repair effect of AM may bring further benefits in improving pathologic conditions.

However, continuous administration is necessary to achieve therapeutic effects because of the conventionally short half-life of peptides in the blood, which can be inconvenient for patients in clinical settings. In this regard, polyethylene glycol (PEG)–modified AM (PEG-AM) is effective in animal models of IBD without compromising the original efficacy of AM, avoiding degradation by peptidases, and extending blood duration by reducing renal clearance.^92^^–^^94^

To our knowledge, this is the first study to investigate the therapeutic efficacy of AM in uveitis. While AM was administered via osmotic pumps, extended half-life formulations such as PEGylated peptides could allow for less frequent dosing, thereby enhancing convenience for patients through regular outpatient injections, similar to other biologics.

In addition, in this study, we adopted a prophylactic dosing approach by starting AM immediately after EAU induction. This experiment was not a therapeutic administration but rather an initial study to evaluate the potential therapeutic effect of AM. While this approach is common in experimental disease models, future studies should evaluate AM's efficacy in advanced stages of uveitis.

In conclusion, we investigated the pathophysiologic significance of the AM-RAMP2 and AM-RAMP3 systems in EAU and clarified the potential mechanism of EAU suppression by the AM-RAMP2 system. AM may exert anti-inflammatory and tissue repair effects by activating Tregs and M2 macrophages; therefore, it has the potential to become a novel therapeutic option for uveitis.

## Supplementary Material

Supplement 1
