# Supplementary material for: Adrenomedullin-RAMP2 System Modulates Inflammation and Tissue Repair in Experimental Autoimmune Uveitis Via T-Cell and M2 Macrophage Regulation
Source: Invest Ophthalmol Vis Sci. 2025 Jun 4;66(6):12. doi: 10.1167/iovs.66.6.12 (PMC12147046; doi:10.1167/iovs.66.6.12)
Supplement: Supplement 1 [file iovs-66-6-12_s001.pdf]

Supplementary Material

Supplementary Methods

Histological Examination

For histological assessment, mice were euthanized by cervical dislocation 14 d post-EAU induction. Eyeballs were removed and fixed in 4% paraformaldehyde to prepare paraffin-embedded sections (5 µm thick) along the papillary-optic nerve plane. Sections were stained with hematoxylin and eosin (HE) and examined using a microscope (BZX710; Keyence, Osaka, Japan). The HE-stained sections were magnified 4× and photographed to include the entire eye. Furthermore, EAU severity was scored for each eye from 0–4, based on previous reports with some modifications (Fig. 1F, Table 2).[55-61] Quantification was performed using a BZ analyzer (Keyence). Additionally, immunohistochemical staining was performed using anti-CD3 (RRID: AB_305055, Cat# ab5690, Abcam, Cambridge, England), anti-F4/80 (RRID: AB_323806, Cat# MCA497GA, Bio-Rad, Hercules, CA) antibodies. Biotin-conjugated secondary antibodies and 3,3′ -diaminobenzidine (DAB) (Histofine kit, Nichirei, Tokyo, Japan) were used to visualize the labeling of CD3, or F4/80. For CD3 or F4/80 staining, the number of positive cells was counted in a 40× field-of-view. For quantification, the number of CD3-positive and F4/80-positive cells in the retina was counted in a section along the papillary-optic nerve plane, as in HE-stained eye sections. When counting, the magnification was increased to 40× and the number of brown-stained cells was summed up as the magnified image was scrolled from one end of the retina to the other. Data were obtained in a blinded manner. All immunohistochemical staining procedures included negative control sections in which the primary antibody was omitted, to verify the specificity of the DAB signal. No background staining was observed in these controls. Isotype-matched control antibodies were not employed.

Quantitative Reverse Transcription Polymerase Chain Reaction

For complementary DNA (cDNA) synthesis, 1 μg of total RNA from the retina or spleen was reverse transcribed using TRI Reagent (Molecular Research Center, Cincinnati, OH, USA) and a RT2 First Strand Kit (Qiagen), following the manufacturer's instructions. Polymerase chain reaction (PCR) primers (Table 3) were designed using NCBI Primer-BLAST (https://www.ncbi.nlm.nih.gov/tools/primer-blast/) and were synthesized by Integrated DNA Technologies (Coralville, IA, USA). Furthermore, quantitative reverse transcriptase (qRT)-PCR was performed using THUNDERBIRD SYBR qPCR Mix (Toyobo) on a StepOnePlus real-time PCR system (Thermo Fisher Scientific). The cycling conditions were 95 °C for 2 min and 40 cycles of 95 °C for 15 s and 60 °C for 1 min, followed by 95 °C for 15 s, 60 °C for 1 min, and 95 °C for 15 s. Relative mRNA levels were normalized to those of mouse glyceraldehyde-3-phosphate dehydrogenase mRNA (Pre-Developed TaqMan assay reagents; Applied Biosystems) and were calculated using the comparative cycle threshold method (ΔΔCt).

Transcriptome Analysis and Data Processing

The transcriptome analysis was conducted using a mouse Clariom S array (Thermo Fisher Scientific). The quantity and quality of RNAs were assessed. All reactions and hybridizations were performed in accordance with the manufacturers' protocols. In brief, biotinylated single-stranded cDNA was generated for hybridization using the GeneChip WT PLUS Reagent Kit (Thermo Fisher Scientific). The arrays were stained using GeneChip Fluidics Station 450 and scanned using the GeneChip scanner 3000 7G system (Thermo Fisher Scientific). The GeneChip Command Console Software ver.3.2 (Thermo Fisher Scientific) was used to process the raw image file data into

intensity data, facilitating gene-level normalization, quality control, and data analysis using the Transcriptome Analysis Console Software ver. 4.0.2 (Thermo Fisher Scientific).

Flow Cytometry

A mixture of Dulbecco's Modified Eagle Medium (Wako), supplemented with 0.5 mg/mL collagenase IV (Sigma-Aldrich, St. Louis, MO) and 50 U/mL DNase I (Wako), was injected into the spleens. The spleens were subsequently minced and digested for 30 min at 37 °C with gentle shaking. Resultant single-cell suspensions were filtered through a cell strainer (100-µm mesh) (AS ONE, Osaka, Japan), resuspended in 1 mL phosphate-buffered saline (PBS) supplemented with 2% fetal bovine serum, and counted using a microscope counting chamber. After excluding dead cells using 0.05% trypan blue, the cell density was adjusted to 5.0×10^6 cells/mL. Next, 1 µL of Fixable Viability Dye 780 (CAS# 67685, Cat# 13-0865-T100) from VERITAS (Silicon Valley, CA) was added per 1 mL of PBS to the cell suspension. This was incubated for 30 min at 4 °C. After cell washing, the supernatant was discarded following centrifugation. Next, the cells were incubated with Fc Block for 10 min at 4 °C, followed by staining with fluorochrome-conjugated antigen-specific antibodies for 20 min at 4 °C in the dark. After cell resuspension in 1 mL of FACS buffer (1× PBS, 1% BSA, 2 nM EDTA, 0.05% NaN_3), 500 µL of Foxp3/Transcription Factor Staining Buffer Set (Cat# 00-5523-00, eBioscience, Carlsbad, CA) for intracellular staining of Foxp3 and CD206. The cells were then incubated at 4 °C in the dark for 60 min. After washing, the cells were centrifuged to remove the supernatant. Next, 500 µL of 1× Permeabilization Buffer (Cat# 00-8333-56) was added in conjunction with Foxp3/Transcription Factor Fixation/Permeabilization Concentrate and Diluent (Cat# 00-5223-56) from eBioscience (Carlsbad, CA), and cells were resuspended in this buffer containing the antibodies of interest. A

final incubation of 60 min at 20–26 °C in the dark was followed by resuspension in FACS buffer and transfer to a glass tube. The reagents and fluorochrome-conjugated antibodies used herein are listed in Table 4. Stained cells were analyzed using a FACS Celesta flow cytometer (BD Bioscience). For each sample, data acquisition was stopped after collecting a total of 100,000 events of live $CD45^+$ cells to ensure consistency across samples. Flow cytometric data were acquired using FACS Celesta (BD Biosciences), and data analysis was performed using Kaluza software (Beckman Coulter, version 2.1.1). Gating was performed as shown in Supplementary Fig. S1A, S2.

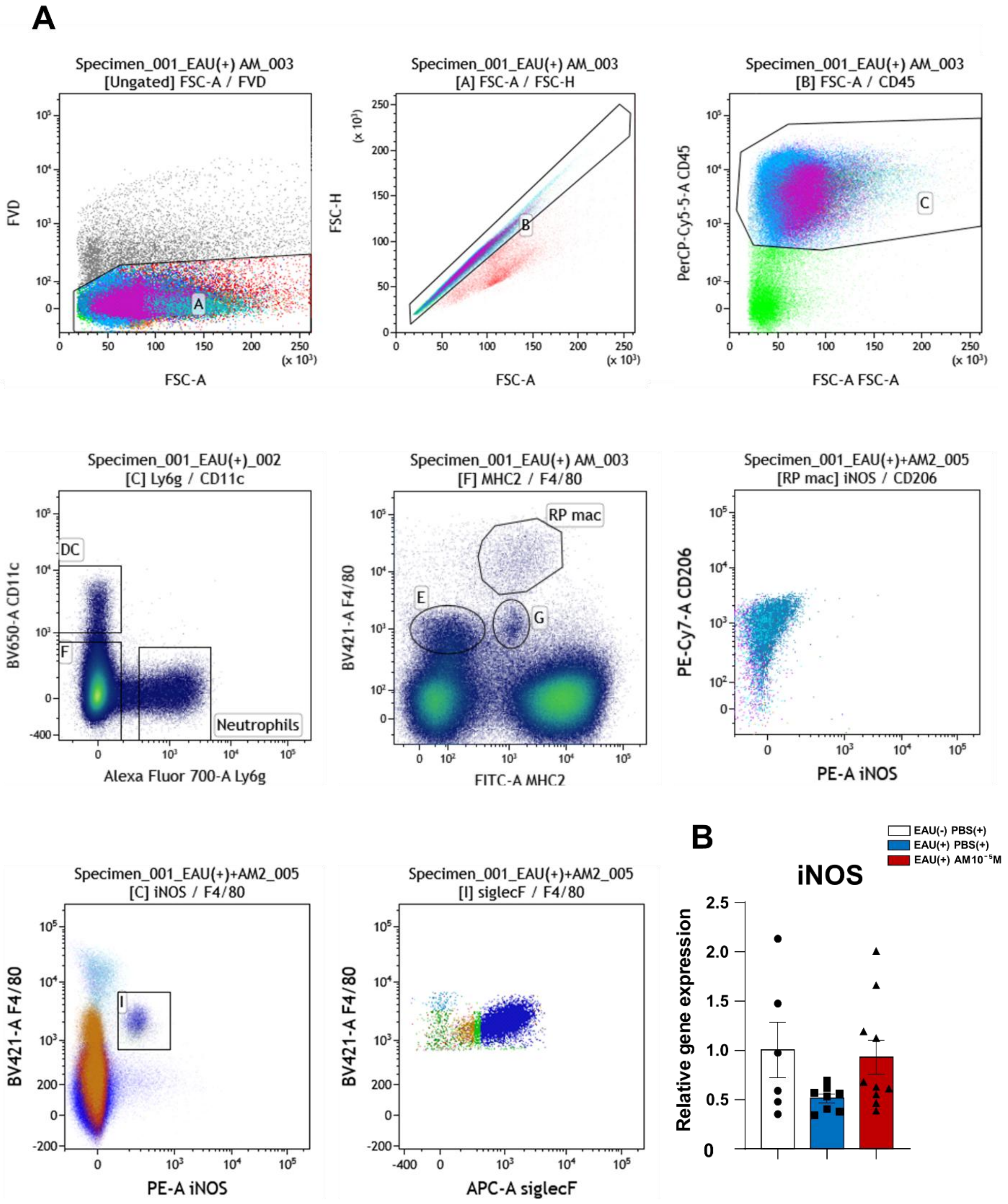

Supplementary Figure 1

Supplementary Fig. S1. Number of iNOS-positive eosinophils activated by interleukin-4 and -13, similar to M2 macrophages, was increased by exogenous adrenomedullin administration in C57BL/6J WT mice

(A) Flow cytometric analysis of spleens in phosphate-buffered saline (PBS)- and adrenomedullin (AM)-treated C57BL/6J mice on day 7 after immunization. Along with gating strategy, Representative scatter plots of dendritic cell (Ly6G-CD11c+ in CD45+), neutrophil (Ly6G+CD11c- in CD45+), red-pulp macrophage (MHC2+F4/80+ in CD45+Ly6G-CD11c-), M2 macrophage (CD206+SiglecF– in CD45+CD11c–Ly6G–), and eosinophil (F4/80- SiglecF+ in CD45+CD11c–Ly6G–) fractions are shown (n=3).

(B) Quantitative reverse transcription polymerase chain reaction (PCR) analysis of iNOS genes in spleens on day 7 after immunization (n=6–10).

AM, adrenomedullin; WT, wild-type; PCR, polymerase chain reaction; iNOS, inducible nitric oxide synthase

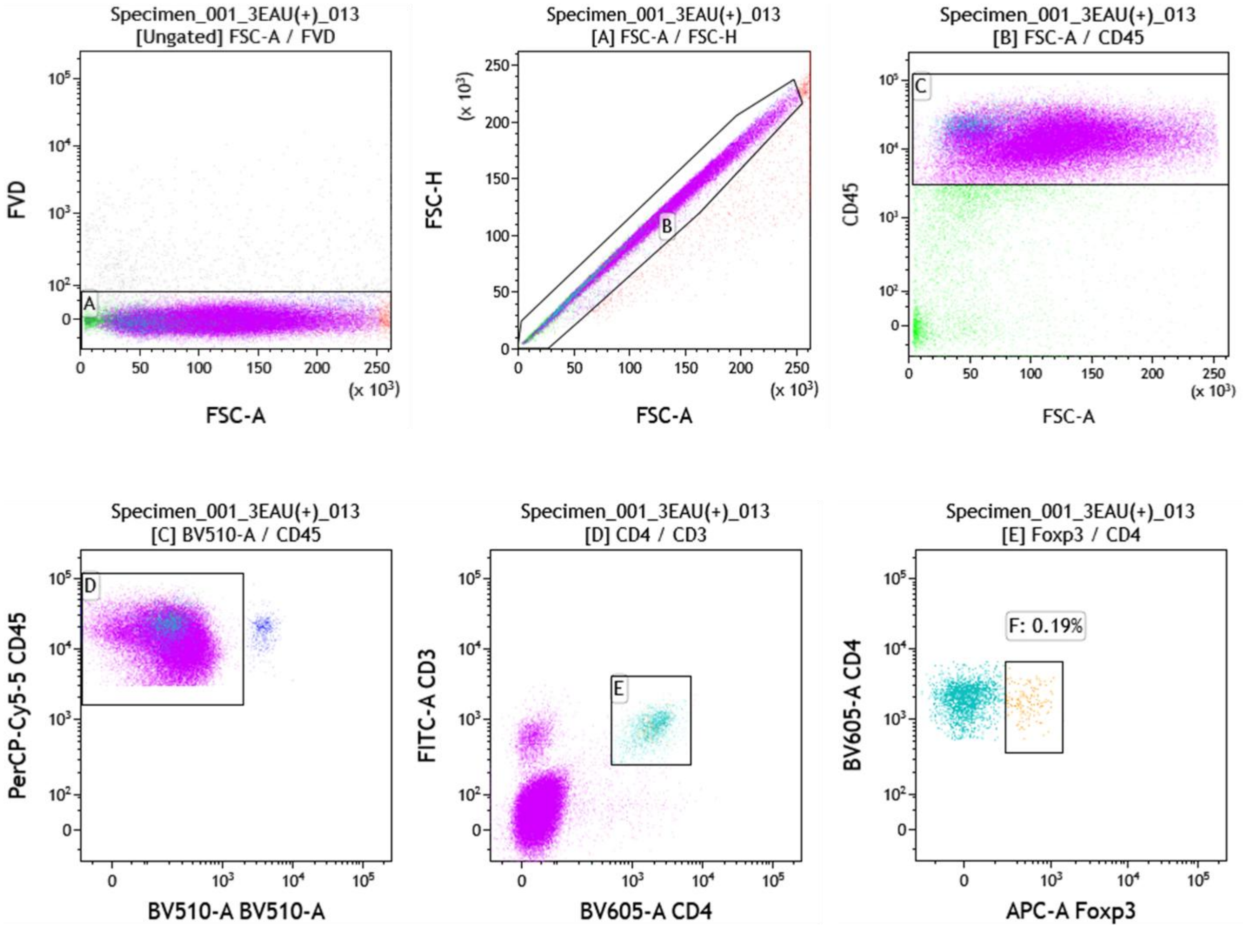

Supplementary Figure 2

Supplementary Fig. S2. Number of CD4- and Foxp3-positive regulatory T cells (Tregs) was increased by exogenous adrenomedullin administration in C57BL/6J WT mice

Flow cytometric analysis of spleens in phosphate-buffered saline (PBS)- and adrenomedullin (AM)-treated C57BL/6J mice on day 7 after immunization. Along with gating strategy, representative scatter plots of T cells (CD3+CD4+ in CD45+) and Tregs (CD4+Foxp3+) fractions are shown (n=3).

AM, adrenomedullin; WT, wild-type

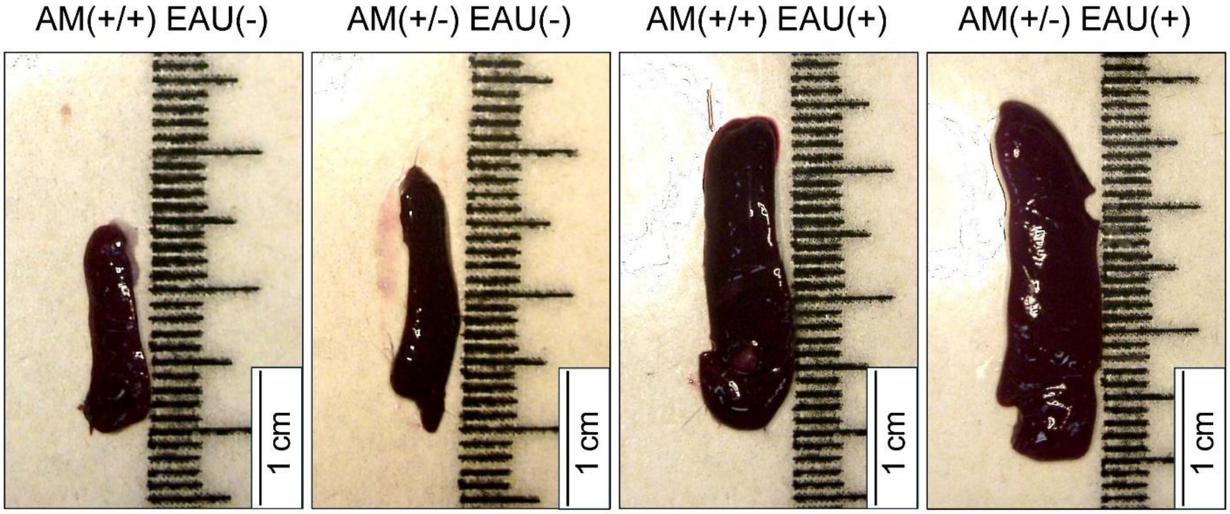

Supplementary Fig. S3. Splenomegaly in experimental autoimmune uveitis was significantly greater in AM (+/-) mice

Splenomegaly and the increase in spleen weight in EAU, is significantly greater in AM (+/-) mice than in AM (+/+) mice. Scale bars: 1 cm.

AM, adrenomedullin; EAU, experimental autoimmune uveitis
